# Anti-Inflammatory and Antiproliferative Properties of Sweet Cherry Phenolic-Rich Extracts

**DOI:** 10.3390/molecules27010268

**Published:** 2022-01-02

**Authors:** Ana C. Gonçalves, Ana R. Costa, José D. Flores-Félix, Amílcar Falcão, Gilberto Alves, Luís R. Silva

**Affiliations:** 1 CICS-UBI—Health Sciences Research Centre, University of Beira Interior, 6201-001 Covilhã, Portugal; anacarolinagoncalves@sapo.pt (A.C.G.); anarfcosta1990@gmail.com (A.R.C.); jdflores@usal.es (J.D.F.-F.); gilberto@fcsaude.ubi.pt (G.A.); 2 CIBIT—Coimbra Institute for Biomedical Imaging and Translational Research, University of Coimbra, 3004-531 Coimbra, Portugal; acfalcao@ff.uc.pt; 3 Laboratory of Pharmacology, Faculty of Pharmacy, University of Coimbra, 3004-531 Coimbra, Portugal; 4 CPIRN-UDI/IPG, Center of Potential and Innovation of Natural Resources, Research Unit for Inland Development (UDI), Polytechnic Institute of Guarda, 6300-559 Guarda, Portugal

**Keywords:** anti-inflammatory, cytotoxicity, oxidative stress, phenolic compounds, sweet cherries

## Abstract

Cherries have largely been investigated due to their high content in phenolics in order to fully explore their health-promoting properties. Therefore, this work aimed to assess, for the first time, the anti-inflammatory potential of phenolic-targeted fractions of the *Saco* cherry, using RAW 264.7 macrophages stimulated with lipopolysaccharide. Additionally, the cytotoxic effects on gastric adenocarcinoma (AGS), neuroblastoma (SH-SY5Y) and normal human dermal fibroblast (NHDF) cells were evaluated, as well as the ability to protect these cellular models against induced oxidative stress. The obtained data revealed that cherry fractions can interfere with cellular nitric oxide (NO) levels by capturing NO radicals and decreasing inducible nitric oxide synthase and cyclooxygenase-2 expression. Furthermore, it was observed that all cherry fractions exhibited dose-dependent cytotoxicity against AGS cells, presenting cytotoxic selectivity for these cancer cells when compared to SH-SY5Y and NHDF cells. Regarding their capacity to protect cancer cells against oxidative injury, in most assays, the total cherry extract was the most effective. Overall, this study reinforces the idea that sweet cherries can be incorporated into new pharmaceutical products, smart foods and nutraceuticals.

## 1. Introduction

Over the past few years, research related to multi-target active compounds, particularly those extracted from natural products, has been widely explored, given their potential for treatment and/or prevention of several disorders [1]. In fact, and in accordance with the most recent reports, almost half of the drugs approved in the last 30 years are derived from nature, mainly from medicinal plants [2]. Even so, and despite their use in traditional medicine, there is still a lack of knowledge about the full biological potential, medicinal value and chemical profile of most natural products.

The genus *Prunus* is distributed worldwide, and includes approximately 430 species; among such species, *Prunus avium*, especially their fruits, known as sweet cherries, have been a target of exhaustive studies [3,4,5,6,7,8]. Since ancient times, their vegetal parts have been used in traditional medicine as diuretics, sedatives, draining and anti-inflammatory agents [9,10]. Supported by scientific evidence, their consumption and economic value are rising worldwide, mostly due to their potential therapeutic properties [11]. These are closely linked to their high content of phenolic compounds, which have already showed potential to counteract oxidative stress and inflammatory conditions [5,9,11,12,13,14,15]. Taking into account that inflammation is considered to be a mechanism of protection against infection or injury, the overexpression of pro-inflammatory enzymes, together with an unbalanced production of free radicals and reactive species, such as nitric oxide (^●^NO) and hydrogen peroxide (H_2_O_2_), induce cell damage and apoptosis, contributing to the development of many chronic inflammatory disorders (e.g., diabetes, obesity and rheumatoid arthritis) [16,17].

Nowadays, it is already accepted that the daily ingestion of phenolic-rich sources is an effective approach to suppress these events, given their capacity to act as antioxidant species, modulate arachidonic acid metabolism (e.g., cyclooxygenase (COX), lipoxygenases and phospholipase A2), interact with pro-inflammatory nuclear factor *κ*B, decrease the expression of inducible nitric oxide synthase (iNOS) and, thus, promote a healthy state [18,19,20,21,22].

Bearing these facts in mind and considering previous studies from our research group, which showed that sweet cherry phenolics possess notable antioxidant and *α*-glucosidase inhibitory effects and the capacity to interfere with human colorectal adenocarcinoma and hepatic cells growth [4,5], we considered it relevant to study, for the first time, the effects of these active metabolites on ^●^NO levels in LPS-stimulated RAW 264.7 macrophages. For this purpose, we prepared three enriched fractions in phenolics extracted from sweet cherries (cv. *Saco*): one rich in coloured phenolics (coloured fraction), another one rich in non-coloured phenolics (non-coloured fraction) and a third one rich in both coloured and non-coloured phenolics (total extract). Additionally, the cytotoxic potential of each fraction against two human cancer cell lines, namely gastric adenocarcinoma (AGS) and neuroblastoma (SH-SY5Y) and the possible mechanisms of action involved were also investigated, as well as their protective effects after exposure to *tert*-butyl hydroperoxide (*t*-BHP), H_2_O_2_, and glutamate. For comparative purposes, we also tested the effects of each fraction on the viability of normal human dermal fibroblast (NHDF) cells.

## 2. Results and Discussion

### 2.1. Anti-Inflammatory Activity

Inflammation is a physiological response of the human body that aims to eliminate, neutralize and/or destroy stimuli resulting from microbial infection or tissue injury [18]. However, when it occurs exaggeratedly, it may become dangerous for host tissues, and may be a precursor of many disorders, including cancer and neurological pathologies [23,24]. In this context, pro-inflammatory COX enzymes convert arachidonic acid into prostaglandins, and higher amounts of tumour necrosis factor (TNF)-*a*, interleukin (IL)-6 and ^●^NO, which are originated from oxygen and l-arginine by inducible nitric oxide synthase (iNOS) [18,25,26]. Furthermore, several pieces of research have been conducted into the effective compounds that can inhibit iNOS, COX-2 and the related pathways, with or without low side effects, in order to prevent the occurrence of chronic disorders. Knowing that many phenolics had already shown to have promising therapeutic applications [12,24,27,28,29], we decided to evaluate the anti-inflammatory potential of the phenolic-targeted fractions from sweet cherries.

#### 2.1.1. Effect of Sweet Cherry Fractions on RAW 264.7 Macrophage Viability

**Figure 1:**
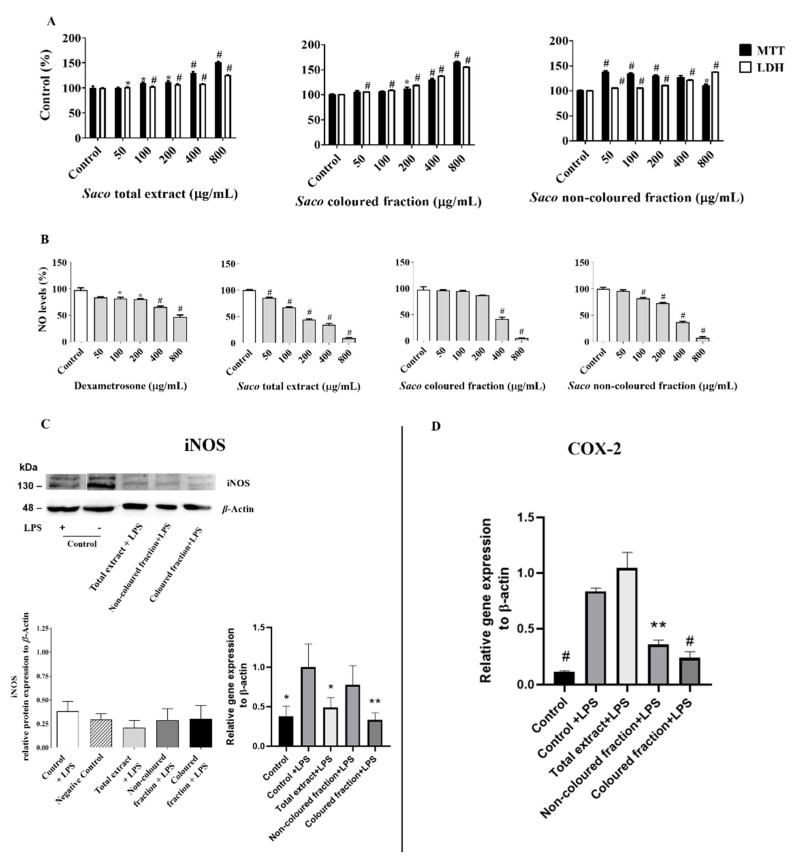
(**A**) Effect of sweet cherry fractions on RAW 264.7 macrophages, evaluated by 3-(4,5-dimethylthiazol-2-yl)-2,5-diphenyltetrazolium bromide (MTT) reduction and lactate dehydrogenase (LDH) leakage assays. (**B**) Effect on nitric oxide levels of cells pre-treated for 2 h with cherry fractions and cherry extract, followed by 22 h co-treatment with 1 µg/mL of lipopolysaccharide (LPS). (**C**) Effect of cherry fractions and cherry extract on LPS-induced iNOS mRNA and protein. The double band at ~130–135 kDa was used to quantify the relative protein expression via Western blot. (**D**) Effect of cherry fractions and cherry extract on LPS-induced COX-2 mRNA expression. *β*-actin was used as an internal control for both Western blot and qPCR analysis. Results are expressed as mean ± SEM of, at least, six independent experiments, performed in triplicate. Statistical differences are shown against LPS-treated controls. * *p* < 0.05, ** *p* < 0.01 and # *p* < 0.0001.

In order to exclude the possibility that the cytotoxicity of phenolic-targeted fractions might contribute to their anti-inflammatory effects on RAW 264.7 cells, preliminary experiments were conducted to assess the range of concentrations for which the exposure to each fraction did not significantly affect cell viability (Figure 1A). Through MTT and LDH leakage assays, it was possible to see that concentrations ranging from 50 to 800 µg/mL did not affect cell viability and, hence, these were chosen for the subsequent experiments.

#### 2.1.2. Effect of Sweet Cherry Fractions on ^●^NO Levels in Cell Culture Medium

Considering the previously selected concentrations, the possible capacity of each fraction to initiate an immune response when in the presence of invaders by decreasing ^●^NO concentrations in the culture medium of LPS-challenged macrophages was evaluated (measured as nitrite formation). The bacterial LPS was used to induce inflammation in these cells and, consequently, to promote the formation of several inflammatory mediators, including ^●^NO and COX-2. Therefore, after 22 h of concomitant exposure to LPS and each fraction from sweet cherries, it was possible to observe a significant concentration-dependent reduction of ^●^NO levels, suggesting the presence of anti-inflammatory effects (Figure 1B). Statistical differences were found between fractions. The total extract was the most effective at scavenging ^●^NO (IC_50_ = 176.29 ± 1.39 µg/mL), followed by the coloured and non-coloured fractions (IC_50_ values of 338.31 ± 1.52 and 367.93 ± 2.10 µg/mL, respectively). All of them were more effective than the positive control, dexamethasone (IC_50_ = 593.64 ± 2.37 µg/mL).

The obtained data are in agreement with other studies focused on the potential of phenolic-rich fractions to reduce ^●^NO levels in culture medium [4,14,26,30]. Furthermore, it was also possible to verify that the combination of coloured and non-coloured phenolic compounds in total extract acts synergistically to enhance the anti-inflammatory potential. Among the phenolics present in cherries, quercetin, *ρ*-coumaric and ferulic acids (at 20 µM), quercetin and kaempferol (at 100 µM) and cyanidin 3-*O*-rutinoside (at 33 µM) already showed the potential to scavenge the ^●^NO produced by LPS-stimulated RAW 264.7 cells [27,28,29,31]. In order to understand if the obtained results can be associated with the capacity of phenolic-targeted fractions to modulate iNOS and/or COX-2, and/or their antioxidant capacity to scavenge ^●^NO, specific studies were conducted, and the results are presented in the following sections (Section 2.1.3 and Section 2.1.4, respectively).

#### 2.1.3. Effect of Sweet Cherry Fractions on LPS-Induced iNOS and COX-2 Expression

Therefore, to expand on the results, and knowing that iNOS and COX-2 are two critical enzymes that exacerbate inflammation, both being the main cells responsible for synthesizing NO and prostaglandins, respectively, we decided to check the capacity of phenolic-target fractions to modulate their expression using Western blot analysis and qPCR. For that, we used protein extracts from cells treated with cherry fractions at 200 µg/mL (coloured fraction) and 400 µg/mL (total extract and non-coloured fraction), which corresponded approximately to almost half of the ^●^NO inhibition. As observed in Figure 1C,D, the incubation of RAW cells under LPS seems to enhance COX-2 and iNOS mRNA expression when compared to untreated cells (negative control). In addition, the treatment with phenolic-target fractions slightly decreases both mRNA expression levels when compared to cells only exposed to LPS alone. Overall, the total extract and coloured fraction seemed to be the most effective at decreasing iNOS expression, while, in COX-2, it was the coloured and non-coloured fractions. Although no statistically significant results were observed in the Western blot assay, significant data were obtained in the qPCR assay that assessed the total extract and coloured fraction for iNOS, and the coloured and non-coloured fractions for COX-2. Similar differences between RNA expression though qPCR and protein detection via Western blot analysis have been reported in other works that have employed LPS as a pro-inflammatory factor. The vestigial increments observed in COX-2 mRNA expression after total extract treatment are considered to be predictable and related to its role of mediating prostaglandin synthesis, which has been verified in the early stages of inflammation [30,32,33]. Additionally, and given the obtained data, it is expectable that this modulation occurs in a concentration-dependent manner and becomes more expressive over time.

The anti-inflammatory effects of sweet cherries are known. For example, Jacob and colleagues [34] already reported that the daily consumption of 280 g of cherries by healthy women can lower plasma C-reactive protein and NO concentrations, 3 h after intake. Additionally, Delgado and collaborators [35] also mentioned the capacity of sweet cherry fruits to down-regulate the levels of IL-1*β* and TNF-*α* pro-inflammatory cytokines and increase IL-4 and IL-2 anti-inflammatory cytokines in rats that consumed 141 g fresh cherries for 10 days.

The anti-inflammatory capacity shown by these fruits is attributed to the presence of anthocyanins. In fact, the anthocyanins extracted from cherries already showed a stronger ability to inhibit COX-2 at a concentration of 125 µg/mL (47.4% of inhibition) than ibuprofen (39.8%) and naproxen (41.3%) [36]. This modulation is due to the capacity of phenolics to down-regulate nuclear factor-kappa B (NF-*κ*B), affecting the biosynthesis of iNOS and COX-2, and consequently reducing the formation of NO and prostaglandins, respectively, and suppressing mitogen-activated protein kinases (MAPKs) and JNK1/phosphorylation [37,38]. Besides the anthocyanins, other phenolics present in cherries also showed the ability to interfere with inflammation-related pathways and reduce pro-inflammatory markers, including hydroxybenzoic acids (25 µM), caffeic acid (10 µM), *ρ*-coumaric acid (50 µM) and quercetin (100 µM) [27,29,39,40,41].

#### 2.1.4. ^●^NO Scavenging Activity

**Figure 2:**
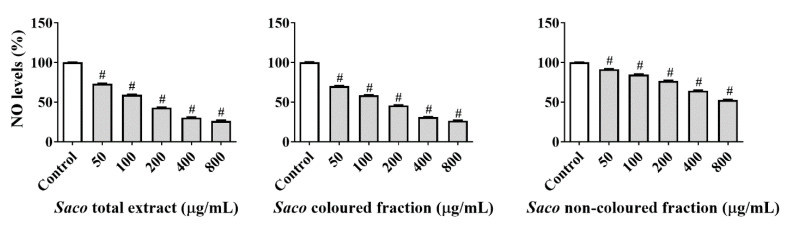
Antioxidant activity of sweet cherry fractions against nitric oxide radicals (^●^NO) in the cell-free assay. Data represent the mean ± SEM of three independent experiments, performed in triplicate (^#^ *p* < 0.001 compared to the respective control).

Taking into consideration the in vitro results obtained with RAW 264.7 macrophage cells, we decided to assess if a process of direct ^●^NO scavenging occurs at the same time, contributing also to the diminishment of the *^●^*NO levels in the culture medium. For this, a cell-free assay based on the photolytic decomposition of sodium nitroprusside was performed, using the same concentrations studied in cells. All the targeted fractions displayed significant scavenging activity in a concentration-dependent manner. The total extract and the coloured fractions were the most active, exhibiting NO reductions of around 26% at the highest concentration tested (800 µg/mL) (IC_50_ values of 156.41 ± 0.96 and 167.29 ± 0.96 µg/mL, respectively) (Figure 2).

In fact, it is well known that the structure of phenolics (and especially the catechol, pyrogallol and methoxy groups) gives them the capacity to transfer hydrogen atoms to radical species and, in this way, diminish their levels [5]. Moreover, the obtained results offer further support for the influence of the interaction of non-coloured phenolics with anthocyanins in the biological potential of sweet cherries. Overall, the results obtained suggest that the decrease in cellular *^●^*NO levels is mainly due to the scavenger capacity of the extracts to scavenge ^●^NO, and less because of their capacity to decrease the expression of iNOS and COX-2.

### 2.2. Effect of Sweet Cherry Fractions on the Viability of Human Cancer Cells

**Figure 3:**
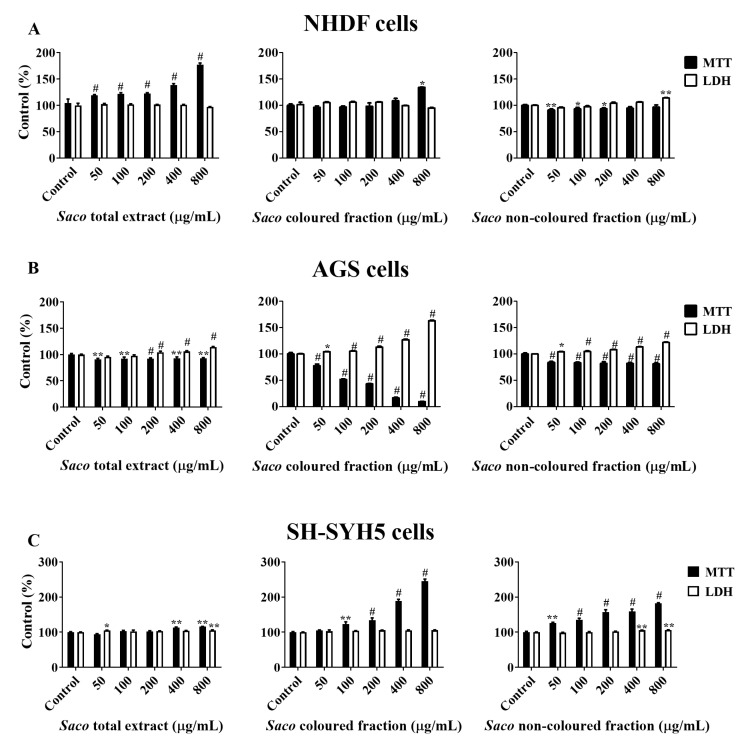
Effects of sweet cherry phenolic-targeted fractions on cellular viability of (**A**) NHDF, (**B**) AGS and (**C**) SH-SYH5 cells, assessed through 3-(4,5-dimethylthiazol-2-yl)-2,5-diphenyltetrazolium bromide (MTT) reduction and lactate dehydrogenase (LDH) leakage assays. Cells were treated with each fraction for 24 h. Values show mean ± SEM of six independent assays performed in triplicate compared to the respective control (* *p* < 0.05, ** *p* < 0.01 and # *p* < 0.0001).

AGS and SH-SY5Y cell lines were selected given that they are largely used as models of cellular response to xenobiotics and dopaminergic cells, respectively [42,43]. In this study, five different concentrations of each fraction (50, 100, 200, 400 and 800 µg/mL) were tested. The MTT assay demonstrated that AGS cells were more sensitive than SH-SY5Y and NHDF cells. In fact, there were verifiably significant decreases in cells viability, in a dose-dependent manner, when AGS cells were incubated with different concentrations of phenolic-targeted fractions from sweet cherries (Figure 3).

The coloured fraction was the most effective at inducing DNA damage and cell death in AGS cells, revealing an IC_50_ of 130.39 ± 1.73 µg/mL. On the other hand, no changes were verified with SH-S5Y5 nor NHDF cells, which supports the cytotoxic selectivity of the fractions for AGS cells (Figure 3). Moreover, and as expected, the most notorious LDH response was also obtained in the highest tested concentrations of the coloured fraction, i.e., 200, 400 and 800 µg/mL, showing values of 112.77, 126.89 and 163.05%, respectively (Figure 3). Since MTT reduction results are more expressive than those of LDH in culture medium, it was also possible to conclude that mitochondrial activity losses happened before the membrane was damaged and, therefore, that the necrotic process only occurs in the highest concentrations tested (400 and 800 µg/mL). This evidence is in agreement with other previous studies [5,43].

Significantly, the obtained data are directly linked to the capacity of phenolics to interact with the different cancer-related pathways, for example, by arresting cell cycles, removing pre-carcinogen agents, regulating metastasis proteins and inducing apoptosis. Additionally, phenolics can also reduce oxidative stress and stimulate DNA repair and, thus, block malignant transformation by promoting cellular differentiation and, consequently, inhibit the development and/or progression of the tumour. These abilities are strongly related to the chemical structure of these compounds, pointing to the carboxyl, hydroxyl and methoxy groups, which promote antioxidant and also pro-oxidant behaviours and anti-inflammatory actions, which in turn, increase their cytotoxicity effects on cancer cells.

In this work, the anticancer bioactivity of cherries is predominantly correlated with anthocyanin content, which is in accordance with previous studies [5,44,45]. In fact, the existence of multiple hydroxyl groups on their B ring enhances their biological potential. In agreement with this observation, it was already reported that the phenolic-enriched fractions obtained from sweet cherries, underlining the anthocyanin-rich fraction, can efficiently interfere with human colon carcinoma Caco-2 cells, exhibiting an IC_50_ of 667.84 µg/mL and a correlation between this activity and an anthocyanin content of 0.6674 [5]. Even so, other non-coloured phenolics present in cherries, e.g., hydroxycinnamic acids, and quercetin derivatives were also revealed to have anti-cancer effects on several human cancer cells, with this activity related to their antioxidative effects [46,47,48].

#### Effect of Sweet Cherry Fractions on the Morphology of AGS Cells

Taking into account the obtained results, and in order to deepen the previous results, morphological and nuclear evaluation assays were also performed.

**Figure 4:**
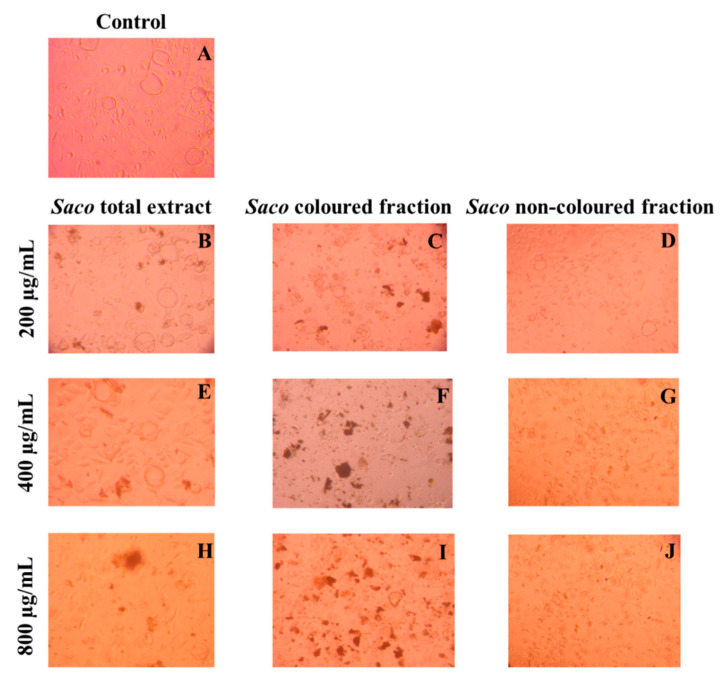
Effects of sweet cherry phenolic-targeted fractions on AGS cells morphology (control vs. treatment after 24 h of incubation). (**A**) Corresponds to the control, (**B**,**E**,**H**) correspond to *Saco* total extract, while (**C**,**F**,**I**) correspond to the coloured fraction and (**D**,**G**,**J**) correspond to the non-coloured one, at concentrations of 200, 400 and 800 µg/mL, respectively. As expected, and considering the data in the previous figure, an increase in debris was observed as the concentration of each fraction increased.

The observation of the cells after treatment under a microscope revealed high amounts of debris (Figure 4), mainly in the highest concentrations (800 µg/mL) (Figure 4H–J), which can be considered to be further evidence regarding the toxicity effects of phenolic-targeted fractions on cancer cells.

**Figure 5:**
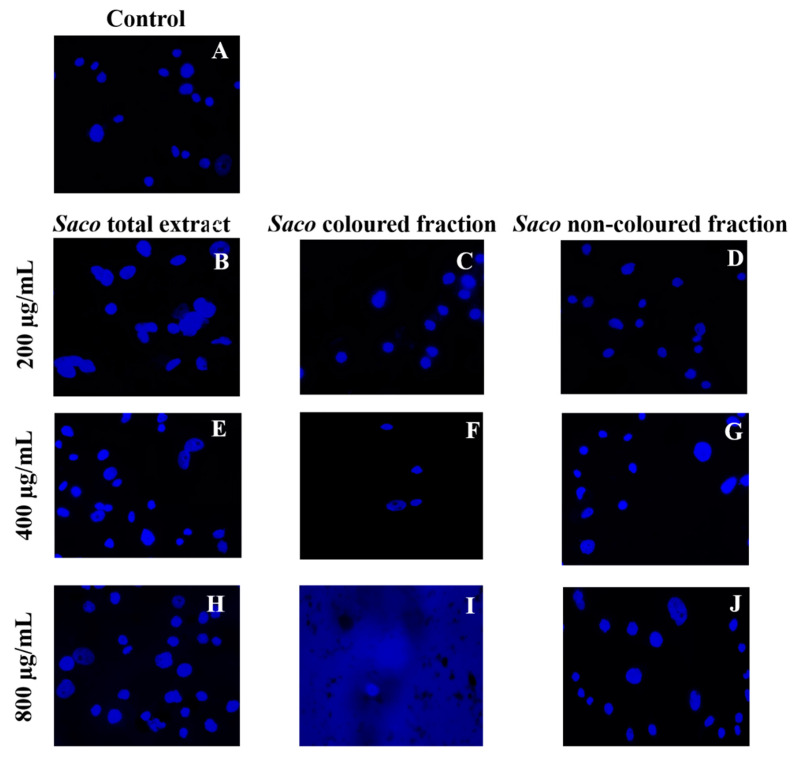
Effects of sweet cherry phenolic-targeted fractions on AGS cell nuclei, visualized with 4′,6-diamidine-2′-phenylindole dihydrochloride (control vs. treatment after 24 h of incubation). (**A**) Corresponds to the control, (**B**,**E**,**H**) correspond to *Saco* total extract, while (**C**,**F**,**I**) correspond to the coloured fraction and (**D**,**G**,**J**) correspond to the non-coloured one, at concentrations of 200, 400 and 800 µg/mL, respectively.

Furthermore, through nuclear staining, it was also possible to clearly observe the formation of cytoplasmatic blebs, followed by cell structure losses, nucleus condensation and vacuolization as the concentration of each fraction increased (Figure 5).

These events are compatible with some types of programmed cell death. As expected, among fractions and in accordance with the obtained values of the viability assays (Figure 3), the most notorious effects were observed for the coloured fraction, where it is possible to see a necrosis event at the highest concentration (800 µg/mL), which is characterized by mitochondrial and cellular swelling following plasma membrane disruption. On the other hand, at 200 µg/mL, we only observed morphological changes, including condensed chromatin and fragmented nuclei, which are characteristics of apoptosis.

Similar results were already reported for other phenolic-rich fractions [5,26,43,49,50]. Particularly, Gonçalves and collaborators [5] revealed that 800 µg/mL of anthocyanin-rich fractions from sweet cherries causes necrosis in Caco-2 cells after 24 h of exposure. Focusing on individual phenolics, Shang and colleagues [51] reported that quercetin at 160 µM causes apoptosis in AGS cells. Furthermore, 100 µM cyanidin 3-*O*-rutinoside and 50 µM of catechin derivatives showed the potential to induce apoptosis in human adenocarcinoma HepG2 cells and breast cancer MDA-MB-231 cells, after 24 and 48 h of exposure, respectively [51,52].

### 2.3. Cytoprotective Effects

The final step of this work was to evaluate the capacity of phenolic-targeted fractions obtained from sweet cherries to protect AGS and SH-SY5Y cells against induced oxidative stress. As is known, oxidative stress plays a crucial role in cancer development and progression and, hence, its relief will interfere with the cancer tumour growth and metastasis. Therefore, it is not surprising that phenolics have been intensively studied in order to discover their full biological potential. Once again, the cellular viability of AGS and SH-SY5Y cells was determined via MTT and LDH leakage assays. The total protection was compared to stressed control cells. In a general way, the obtained outcome is very promising and revealed that phenolics can protect against oxidative stress and apoptosis.

**Figure 6:**
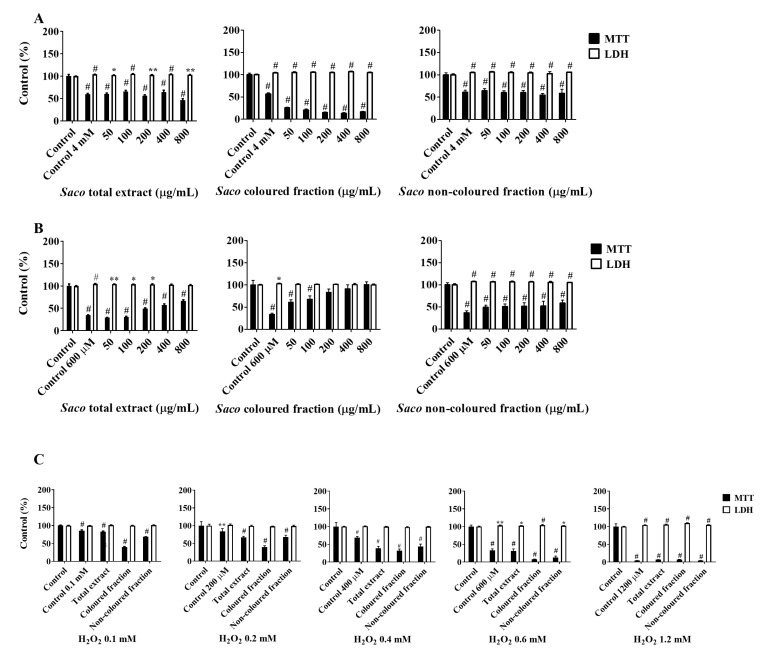
Effects of sweet cherry phenolic-targeted fractions on cellular viability of AGS cells, assessed through 3-(4,5-dimethylthiazol-2-yl)-2,5-diphenyltetrazolium bromide (MTT) reduction and lactate dehydrogenase (LDH) leakage assays, after exposure to *Saco* fractions for 24 h, and then insulted with (**A**) *tert*-butyl hydroperoxide (4 mM; 2 h) and (**B**) hydrogen peroxide (H_2_O_2_; 600 µM; 2 h). (**C**) Additionally, and after treatment using the non-toxic concentration of 50 µg/mL of each fraction, cells were also exposed to different concentrations of H_2_O_2_ (100, 200, 400, 600 and 1200 µM) for 24 h. Values show mean ± SEM of six independent assays performed in triplicate compared to the respective negative control (* *p* < 0.05, ** *p* < 0.01 and # *p* < 0.0001).

Firstly, the capacity of phenolics to protect AGS cells after exposure to *t*-BHP and H_2_O_2_ was assessed. In most assays, the total protection was accomplished in the lowest tested concentrations (50 and 100 µg/mL). Regarding the protection offered by phenolics against *t*-BHP (Figure 6A), we observed that the non-coloured fraction was the most effective, showing increments in cell viability rates of 3.42% at 50 µg/mL. On the other hand, neither the coloured fraction concentration nor the concentrations of 200 and 800 µg/mL of the total extract showed the capacity to protect these cells against *t*-BHP-induced oxidative stress. Even so, dose-dependent protection was observed in AGS cells against the oxidative damage induced by 600 µM H_2_O_2_ (Figure 6B). Amongst the fractions, the coloured fraction showed the highest protection, revealing increments of viability between 27.10 and 67.17%, followed by the total extract and non-coloured fraction, which can be considered to be further evidence of the strong antioxidant effects shown by anthocyanins. Regarding the insult with different concentrations of H_2_O_2_ for 24 h (Figure 6C), none of the fractions nor the total extract showed the capacity to protect these cells against the induced oxidative stress.

**Figure 7:**
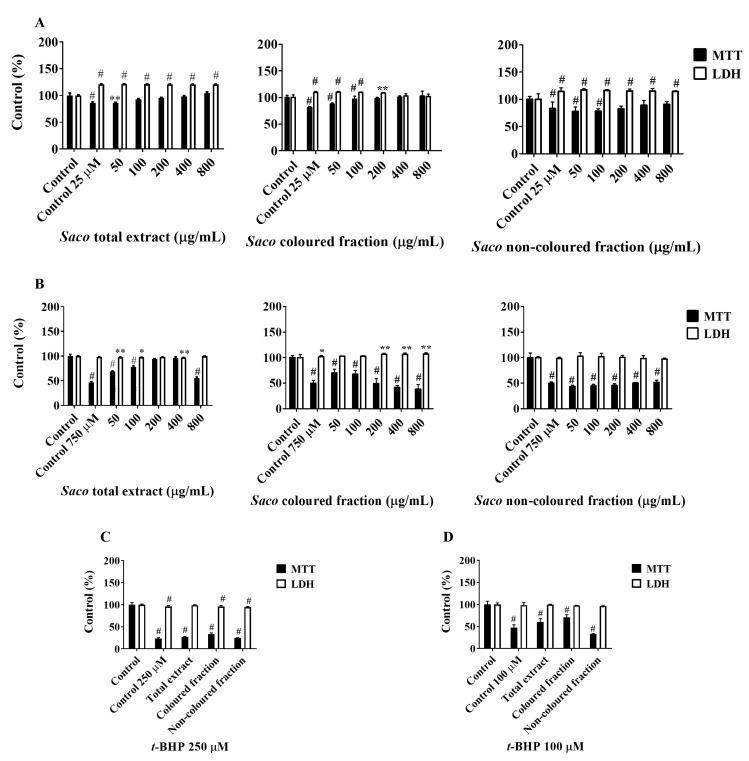
Effects of sweet cherry phenolic-targeted fractions on cellular viability of SH-SY5Y cells, assessed through 3-(4,5-dimethylthiazol-2-yl)-2,5-diphenyltetrazolium bromide (MTT) reduction and lactate dehydrogenase (LDH) leakage assays, after exposure to *Saco* fractions for 24 h and then insulted with (**A**) glutamate (25 µM; 6 h) and (**B**) hydrogen peroxide (750 µM; 24 h). Additionally, and after treatment using the non-toxic concentration of 50 µg/mL of each fraction for 6 and 24 h, cells were insulted with *tert*-butyl hydroperoxide (*t*-BHP) for a further 12 and 24 h, at concentrations of (**C**) 250 or (**D**) 100 µM, respectively. Values show mean ± SEM of six independent assays performed in triplicate compared to the respective negative control (* *p* < 0.05, ** *p* < 0.01 and # *p* < 0.0001).

In SH-S5Y5, all targeted fractions showed effectiveness at protecting these cells against the neurotoxicity induced by glutamate in a dose-dependent manner. The total protection was again achieved in the lowest tested concentrations (50 and 100 µg/mL). Unsurprisingly, the total extract was the most promising one, promoting rises in cell viability ranging from 0.34 and 18.44%, which suggests that the combination of different phenolics is an added value in intensifying the health benefits (Figure 7A).

Regarding the protective effects offered by phenolics against the induced oxidative stress promoted by H_2_O_2,_ the coloured fraction revealed pro-oxidant behaviour in the highest tested concentrations (100–800 µg/mL). On the contrary, the non-coloured extract in all concentrations and the total extract (≤400 µg/mL) showed the capacity to protect these cells in a dose-dependent manner (Figure 7B). Furthermore, the phenolic-target fractions also presented the potential to protect neuronal cells after exposure to *t*-BHP at different concentrations and times (Figure 7C,D). In both experiments, the coloured fraction was the most notorious for attenuating the *t*-BHP-induced cytotoxicity, followed by the total extract and non-coloured fraction. The obtained results revealed that the capacity of phenolics to protect against oxidative injury increases with the time of exposition, and also with the concentration of the pro-oxidant agent.

Overall, the protection showed by phenolics is, in part, mediated by antioxidant mechanisms. Furthermore, the interactions occurring between different phenolic subclasses also serve to increase their biological potential. Indeed, it was already documented that phenolics can pass through the cellular membrane and, hence, scavenging the radicals before them can cause damage in cells and promote apoptosis [5,12,43]. Regarding individual compounds, Vepsäläinen and collaborators [53] already reported that quercetin and anthocyanin-rich extracts from berries (0.25 to 31 µg/mL) can significantly decrease reactive oxygen species production on neuroblastoma cells (46% and 86%) in a dose-dependent manner. Other phenolics, including phenolic acids, also showed the ability to attenuate oxidative stress in cancer cells [5,54,55,56]. Even so, it is also important to note that these effects are strongly dependent on the time and concentration of the insulting agent. Furthermore, it is also important to take into account that, in some situations, multiple substitutions by hydroxyl groups in the structure of phenolics can result in pro-oxidant effects, which, in turn, serve to enhance cellular reactive species concentrations with the objective of intensifying their cytotoxic levels and suppressing cancer cell growth [4,57,58,59]. Of course, these pro-oxidant behaviours are also dependent on the concentrations used [60].

## 3. Materials and Methods

### 3.1. Reagents

All chemicals used were of an analytical grade and were used as received without any further purification unless otherwise specified. N-(1-naphthyl)ethylenediamine dihydrochloride, sulfanilamide and sodium nitroprusside dihydrate (SNP) were purchased from Alfa Aesar (Karlsruhe, Germany). Dulbecco’s Modified Eagle Medium with GlutaMAX™ supplement (DMEM + GlutaMAX), iNOS primary antibody and anti-rabbit HRP-conjugated secondary antibody were obtained from Invitrogen (Grand Island, NY, USA). Foetal bovine serum, antibiotics (10,000 U/mL penicillin, 10,000 mg/mL streptomycin), trypsin-ethylenediaminetetraacetic acid solution, 3-(4,5-dimethylthiazol-2-yl)-2,5-diphenyltetrazolium bromide (MTT), dimethyl sulfoxide (DMSO), dexamethasone and *β*-nicotinamide adenine dinucleotide (NADH) were from Sigma-Aldrich (St. Louis, MO, USA). Water was deionized using a Milli-Q water purification system (Millipore Ibérica, Madrid, Spain).

### 3.2. Samples

Approximately 1 kg of *Saco* sweet cherry fruits, grown in the Fundão region (Portugal), was harvested by hand in June 2017, at the commercial maturity stage. Within 1 h of harvest, samples were transported to the laboratory facilities at 0 °C. Then, their pits were removed and separated from the pulp, which, in turn, was frozen with liquid nitrogen and maintained at −80 °C until lyophilization. After lyophilization, the pulp was powdered and divided into the three aliquots used for the preparation of the extracts.

### 3.3. Extract Preparation

The preparation of the cherry extracts was performed according to a previous method [5]. Briefly, 1 g of powered cherries was extracted with 20 mL ethanol 70% for 2 h, under agitation at room temperature, and protected from light. Then, the obtained homogenates were centrifuged at 2900× *g* for 10 min. After that time, the supernatant, i.e., the solvent cherry extract, was collected and evaporated under reduced pressure at 30 °C. In order to obtain the fractions enriched in phenolic compounds, a solid-phase extraction (SPE) procedure was performed using Sep-Pak C18 solid-phase extraction columns (70 mL/10,000 mg; Macherey-Nagel, Düren, Germany). The resulting extract was dissolved in 50 mL deionized water and placed in the SPE cartridge preconditioned with 20 mL ethyl acetate, 20 mL ethanol and 20 mL 0.01 mol/L HCl. The loaded cartridge was again washed with 3 mL 0.01 mol/L HCl. The fraction enriched with non-coloured phenolics (fraction I) was eluted with 20 mL ethyl acetate, while the fraction with anthocyanins (fraction II) was eluted with 40 mL ethanol containing 0.1% HCl. To obtain the combined extract (fraction III), another SPE column was performed, being preconditioned as previously described, then the extract was passed through the column and eluted with 40 mL ethanol containing 0.1% HCl. Next, each eluate was concentrated under reduced pressure, and the obtained residues were dissolved in deionized water and lyophilized. Finally, they were stored in silica at room temperature, and protected from light, until their use.

The phenolic profile of each fraction was already analysed via chromatographic techniques. Among the phenolics, hydroxycinnamic acids were the main phenolic compounds found in non-coloured fraction (99.7%) and total extract (69.8%), while cyanidin 3-*O*-rutinoside was the predominant anthocyanin in the coloured fraction (81.5%) and total extract (24.5%) [5].

### 3.4. Cell Models

AGS cells were acquired from Sigma-Aldrich (St. Louis, MO, USA), and NHDF cells from the American Type Culture Collection (LGC Standards S.L.U., Barcelona, Spain). SH-SY5Y and RAW cells were kindly provided by colleagues from CICS-UBI (Covilhã, Portugal). AGS and SH-SY5Y cells were cultured in DMEM + GlutaMAX, while RAW and NHDF cells were maintained with a DMEM and RPMI 1640 medium supplemented with 2 mM L-glutamine, 10 mM 4-(2-hydroxyethyl)-1-piperazineethanesulfonic acid and 1 mM sodium pyruvate, respectively. All mediums were supplemented with 10% foetal bovine serum and 1% penicillin/streptomycin, and maintained in a humidified atmosphere of 5% CO_2_, at 37 °C.

After a few passages, and in order to evaluate the cytotoxic and pro-apoptotic effects of the cherry fractions on RAW 246.7 macrophages, AGS, SH-SY5Y and NHDF cells, they were seeded in 96-well plates at a density of 2.5 × 10^4^, 1.0 × 10^4^, 3.0 × 10^4^ and 1.0 × 10^4^ cells per mL, respectively. After 24 h, the medium was removed, and different concentrations of cherry fractions (ranging from 50–800 µg/mL) dissolved in the medium containing 0.5% (*v/v*) DMSO were added, and plates were incubated again for another 24 h [26,50].

To evaluate the cytoprotective effects of cherry fractions on cells, preliminary assays were performed to determine the appropriate concentration and exposure time of each oxidative stress inducer able to cause around 50% cell death (data not shown). Therefore, in AGS cells, 24 h after the exposure with the fractions, the medium was removed, and cells were exposed to *t*-BHP (4 mM; 2 h) or H_2_O_2_ (600 µM; 2 h). Additionally, and after treatment using the non-toxic concentration of 50 µg/mL of each fraction, cells were also exposed to different concentrations of H_2_O_2_ (100, 200, 400, 600 and 1200 µM) for 24 h. On the other hand, SH-SY5Y cells were exposed to glutamate (25 µM; 6 h), H_2_O_2_ (750 µM; 24 h) or *t*-BHP (100 µM; 24 h) after 24 h of treatment with each fraction [26]. To deepen the outcome, SH-SY5Y cells were also exposed to 250 µM *t*-BHP for 12 h, after 6 h of treatment with each fraction.

All experiments were conducted in the cells’ logarithmic growth phase. Results are expressed as percentage of the respective control and correspond to the mean ± standard error of the mean (SEM) of, at least, six independent experiments performed in triplicate.

#### 3.4.1. Membrane Integrity Assay

The release of the stable cytosolic enzyme lactate dehydrogenase (LDH) into the medium is used as a marker for loss of membrane integrity, and it can be assessed spectrophotometrically at 340 nm (Bio-Rad Laboratories, Hercules, CA, USA) in a kinetic mode. It is based on the conversion of pyruvate to lactate by LDH, using NADH as a cofactor [5]. Briefly, after each assay, 50 µL of culture medium was placed in 96-well plates and mixed with NADH (252.84 mM) and pyruvate (14.99 mM). Both pyruvate and NADH solutions were prepared in phosphate-buffered saline (PBS; pH 7.4). A decrease in absorbance is directly related to the decrease in NADH levels. Untreated cells were used as a control.

#### 3.4.2. MTT Reduction Assay

Cell viability was determined using the colorimetric 3-(4,5-dimethylthiazol-2-yl)-2,5-diphenyltetrazolium bromide (MTT) assay. To accomplish this, at the end of each experiment, the medium was removed and MTT (0.5 mg/mL dissolved in the appropriate serum-free medium) was added and incubated at 37 °C for 4 h. Afterwards, MTT was discarded, and the formazan crystals were solubilized using DMSO. The absorbance was read at 570 nm using a microplate reader, the Bio-Rad Xmark spectrophotometer. Untreated cells were used as a control.

### 3.5. Intracellular Polyphenol Staining and Fluorescence Microscopy

The morphological studies were based on previous work [50]. Briefly, AGS cells were seeded at a density of 7.5 × 10^4^ cells per mL, in coverslips placed in 24-multiwell plates. After 24 h, the medium was discarded, and the adherent cells were treated with different concentrations of each fraction (50–800 µg/mL) for another equal period of time. Next, the medium was removed, and cells were carefully washed with PBS and then fixed in coverslips with 4% of paraformaldehyde solution prepared in PBS, followed by 10 min of incubation at room temperature. Then, the solution was rejected, and the fixed cells were again repeatedly rinsed with PBS. Nuclear morphology was observed using 4,6-diamidino-2-phenylindole (DAPI), added to the fixed cells at 1 µg/mL for 10 min at room temperature. Finally, cells were washed twice with PBS and chromatin fluorescence was analysed using a Zeiss AxioImager A1 fluorescence microscope. Digital images were generated with AxioVision 4.8.2 software.

### 3.6. Determination of ^●^NO Levels in Culture Medium Interference

The nitrite accumulation in the culture medium was determined according to a method described by Taciak and colleagues [61]. Cells were cultured at density of 15 × 10^4^ cells per mL in 96-well plates for 24 h at 37 °C and 5% CO_2_. Then, the medium was removed, and cells were exposed to increasing concentrations of each fraction for 2 h. After that period, cells were stimulated with 1 µg/mL LPS for a further 22 h. The nitrite conversion was determined using a mixture composed of 75 µL of culture media mixed with an equal volume of Griess reagent (1% sulphanilamide and 0.1% N-[naphth-1-yl]ethylenediamine dihydrochloride in 2% H_3_PO_4_), after an incubation period of 10 min, in the dark, at room temperature. The absorbance was then measured at 560 nm in a microplate reader (Bio-Rad Laboratories, Hercules, USA). ^●^NO levels were expressed as a percentage of the ^●^NO in cells exposed to LPS (positive control) and correspond to the mean ± SEM of six independent experiments, performed in triplicate. Dexamethasone at equal concentrations of each tested concentration was used as a positive control.

### 3.7. Detection of Inducible Nitric Oxide Synthase (iNOS) Expression

Western blot analysis was carried out with protein extracts obtained from RAW 264.7 cells based on the method reported by Pereira and colleagues [62], with some modifications. Briefly, RAW 264.7 cells were cultured in six-well plates at density of 50 × 10^4^ for 24 h. Then, the medium was removed, and the cells were exposed to each fraction for 2 h, followed by the addition of 1 µg/mL LPS for further 24 h. Afterwards, cells were washed with PBS, scraped, and incubated on ice with ice-cold RIPA lysis buffer (150 mM NaCl, 0.5% sodium deoxycholate, 0.1% SDS, 1% Triton X-100, 50 mM Tris pH 8.0, 1 mM PMSF, 1 mM sodium orthovanadate and 40 µL/mL of complete EDTA-free protease inhibitor cocktail) for 30 min. Then, cell debris were removed by microcentrifugation (10,000× g for 10 min). Total protein content was measured using a Pierce BCA Protein Assay Kit (Thermo Fisher Scientific, Waltham, MA, USA) according to the manufacturer’s recommendations. After quantification, 30 µg of total protein was mixed with a loading buffer containing 4% *β*-mercaptoethanol, followed by denaturation for 5 min at 100 °C, and then loaded in 8% or 12.5% SDS-PAGE. Proteins were subsequently electrically transferred onto polyvinylidene difluoride membranes (Millipore, Merck, Milford, CT, USA), using a Trans-Blot^®^ Cell system (Bio-Rad, Hercules, CA, USA). Next, membranes were blocked with a solution of 5% skimmed milk powder in Tris-buffered saline (TBS), for 1 h at room temperature, and incubated overnight at 4 °C with primary antibody rabbit anti-iNOS (1:300). After, membranes were washed at room temperature with TBS containing 0.1% of Tween and incubated for 1 h at room temperature with the respective HRP-conjugated secondary antibody (anti-rabbit 1:20,000). Then, membranes were washed, and antibody binding was detected using the SuperSignal™ West Pico PLUS Chemiluminescent Substrate (ThermoFisher Scientific, Grand Island, NE, USA) according to the manufacturer’s instructions. Images of blots were captured with the ChemiDoc MP Imaging system (Bio-Rad, Hercules, CA, USA). Additionally, the expression of iNOS was normalized with *β*-actin (loading control). Then, blots were incubated for 2 h at room temperature with mouse anti-*β*-actin (1:20,000) before incubation for 1 h with HRP-conjugated goat anti-mouse secondary antibody (1:40,000). Next, images were acquired, and protein bands were quantified using the Image Lab software (Bio-Rad, Hercules, CA, USA). The results correspond to the mean ± SEM of, at least, four independent experiments.

### 3.8. iNOS and Cyclooxygenase (COX)-2 Gene Expression Analyses

The quantitative analysis regarding iNOS and COX-2 gene expression via qPCR was conducted under similar conditions to those applied to perform the Western blot assay. The cells were harvested and total cellular RNA was extracted using the NZY total RNA Isolation kit (NZYTech, Lisboa, Portugal) according to the manufacturer’s instructions. For each RT-PCR reaction, the total RNA was loaded in a One-step NZYSpeedy RT-qPCR Probe kit (NZYTech, Lisboa, Portugal) to carry out the synthesis of cDNA, and the PCR reaction was completed in a CFX Connect Real-Time PCR Detection System (BIO-RAD, Hercules, CA, USA). *β*-actin was employed as an internal control. The oligonucleotide primers used were: 5′-GAGCGAGTTGTGGATTGTC-3′ (forward) and 5′-CTCCTTTGAGCCCTTTGT-3′ (reward) for iNOS; 5′-GGAGAGACTATCAAGATAGT-3′ (forward) and 5′-ATGGTCAGTAGACTTTTACA-3′ (reward) for COX-2; 5′-CTGTCCCTGTATGCCTCTG (forward) and 5′-ATGTCACGCACGATTTCC-3′ (reward) for *β*-actin. The thermal cycling conditions were as follows: 20 min at 50 °C for cDNA synthesis, 2 min at 95 °C for retrotranscriptase inactivation, followed by 40 cycles of denaturation at 95 °C for 5 s and annealing/extension at 55 °C for 1 min. The fluorescence signal was detected at the end of each cycle. The results were analysed with BIORAD CFX Manager 3.1 (BIO-RAD, Hercules, CA, USA), and a melting curve was used to confirm the specificity of the products. The expression levels of the target genes were normalized to the reference gene *β*-actin. At least three independent experiments were performed and all reactions were completed in duplicate to confirm reproducibility.

### 3.9. Determination of ^●^NO Levels in Cell-Free System

The capacity of sweet cherry extracts to capture ^●^NO was based on the work of Gonçalves et al. [5]. Briefly, five different concentrations equal to the ones tested in cells were dissolved in potassium phosphate buffer (100 mM, pH 7.4), and mixed with 100 µL SNP (20 mM). The blank and control contained 100 µL phosphate buffer and 100 µL SNP. Then, the plates were incubated at room temperature for 1 h, under light. Subsequently, an equal volume of Griess reagent (1% sulfanilamide and 0.1% naphthylethylenediamine in 2% H_3_PO_4_) was added to each well, and plates were incubated for 10 min in the dark (blanks received 100 µL of H_3_PO_4_). After that time, the absorbance was recorded at 560 nm. The ^●^NO scavenging activity was determined through the comparison of the absorbances between the extracts and the control and corresponded to the mean ± SEM of three independent experiments, performed in triplicate.

### 3.10. Statistical Analysis of Results

Statistical analysis was performed using GraphPad Prism Version 6.01 (San Diego, CA, USA). A one-way ANOVA followed by Dunnett’s post hoc test (LDH and MTT assays) were used to determine the statistical significance in comparison to the control. Values of *p* < 0.05 were considered to be statistically significant.

## 4. Conclusions

Considering the current interest in cherry fruits given their high content of phenolic compounds as functional foods, the obtained data revealed that phenolic-targeted fractions from sweet cherries can exert anti-inflammatory and antiproliferative properties on RAW macrophages and AGS cells, respectively, and also have the capacity to counteract oxidative stress in cancer cells. Additionally, anthocyanins and non-coloured phenolics seem to act synergistically, which may contribute to the health-promoting properties attributed to sweet cherries. Altogether, this work supports their incorporation into pharmaceutical products, nutraceuticals and dietary supplements, once phenolics can be considered to be promising agents in the prevention and/or treatment of diseases mediated by inflammatory mediators, reactive species and free radicals. This notwithstanding, to exclude the risk of toxicity and demonstrate their safety, clinical trials should be conducted to explore the full biological potential of sweet cherries and their safe dosage.

## Data Availability

Data are contained within this article.
